# The role of early inter-professional and inter-agency encounters in increasing students’ awareness of the clinical and community context of medicine

**DOI:** 10.1007/s40037-016-0285-1

**Published:** 2016-07-18

**Authors:** Charankumal Singh Thandi, Simon Forrest, Catherine Williamson

**Affiliations:** School of Medicine Pharmacy and Health, Queens Campus, Durham University, Stockton-On-Tees, UK

**Keywords:** Inter-disciplinary, Multi-disciplinary, Learning, Students, Clinical, Community

## Abstract

Coordinated input from a variety of health and social care professionals into medical education helps students to become strong, effective, successful and competent future practitioners able to function within the multi-disciplinary environment which characterizes modern medicine. This paper presents a new model of teaching developed within the context of the Phase 1 Medicine Programme at Durham, which has been used to help prepare students for this by intertwining a selection of lectures and activities run by external organizations with additional clinical exposure and experience. This one-week learning journey was called the Additional Clinical Experience (ACE) week, and now forms an integral part of the curriculum at Durham University.

## Background

The modern doctor is very much part of a healthcare team and structured exposure to and engagement with other professionals during their training not only promotes medical students’ confidence and competency to collaborate with other practitioners by recognizing their views, values and skills, but also enhances mutual understanding that can lead to improvements in the delivery of service, patient safety and quality of care [1, 2, 3]. The importance of this is recognized in guidance on medical education handed down by the profession’s regulatory body in the UK, the General Medical Council (GMC) [4, 5], which emphasizes amongst expected learning outcomes for graduates the importance of teamwork, communication and working collaboratively with colleagues whilst respecting their skills and contributions.

Additional Clinical Experience (ACE) week was introduced into the Phase 1 Medicine programme at Durham University in 2012 to enhance students’ ability to meet these learning outcomes by providing them with multiple opportunities to engage with a wide variety of healthcare practitioners and services in a variety of contexts and to develop their clinical and communication skills.

ACE builds on the ethos of the Durham programme with its strong orientation and basis in early clinical and community exposure. Throughout the programme students are given opportunities to make visits to hospitals, general practices and organizations working in community health and development. Many lectures provided on campus include patients who present on their experiences of health and ill-health and their contribution is supplemented by lectures and small-group work run by senior hospital clinicians and general practitioners. These contributions to the curriculum have always been highly regarded by students and ACE week represented an attempt to increase their presence and also students’ capacity to elect with which issues, specialties and professionals to engage with by creating a flexible space in the timetable. This paper focuses on ACE week sketches in a description of its format and content, provides some evaluative data and reflections on its impact on students, particularly as it helps to meet the recommendation that early introduction of inter-professional education in the first two years of medical education is likely to be effective [6, 7].

## ACE week

ACE week consists of a non-timetabled week within the busy undergraduate curriculum during which students select from a menu of on- and off-campus learning activities. Off campus there are many opportunities to learn through visits to community services ranging from public services to charitable organizations working in health and social care to hospices and hospitals.

On-campus activities include presentations from keynote speakers, workshops led by patient groups, voluntary sector organizations and healthcare professionals from a broad range of contexts and clinical specialties, and some practical sessions in which students experience working in teams. Examples of the kinds of activities on offer to students include the following:

*Journey through the asylum-seeking process:* A workshop looking at the effect of past experiences and future fears on the well-being of those looking for sanctuary in the UK. This session was run by Justice First, a local charity providing advice, advocacy and support to refugees and asylum-seekers.

*HIV & sexual health workshop:* A session with a local HIV/Aids organization covering routes and risks of transmission, sexual activity, sexual language and working with patients. This was offered by Teesside Positive Action, a local charitable organization providing sexual health services for the community.

*Journey from birth to hospice:* The experience of the child and family of hospice care. This session focused on the roles of the multi-professional team at the Baby hospice, a charity providing respite and palliative care services for children.

*Simulation-based learning:* An opportunity for students to investigate a cardio-respiratory case related to cardiovascular respiratory and renal medicine, using SIMMAN. This session was facilitated by a physiologist and senior lecturers from Durham Medical School.

*Opportunities in medicine – an African adventure:* A personal reflection from a consultant paediatrician on the challenges and experience of two years spent doing voluntary medical work in Tanzania.

*Beautiful boy:* A talk about Down’s syndrome from a patient. This session was provided by a charity, the Education Centre for Children with Downs Syndrome and a patient.

## The development of ACE week: a partnership approach

Since its establishment, student involvement with ACE week has progressively increased. Student feedback was used early on within the ACE week planning group to help shape the structure and type of activities. Where possible, specific requests for activities or workshops were programmed into the subsequent ACE weeks. Since 2014 students have taken the initiative to organize workshops and become involved in peer-to-peer teaching. Many students attributed their enthusiasm to share their experiences to the success of previous ACE weeks, on the basis of which they could see the potential benefit to their and their peers’ development towards becoming more holistic and community-focused doctors.

## ACE week evaluation

ACE week has been evaluated by gathering feedback from both students and individuals and organizations that offered sessions and activities. This has been consistently positive.

Feedback from providers of sessions has been characterized by some consistent themes. Those offering opportunities for day-long experiences off campus often point to the value of being able to show a student a more rounded picture of the organization. For example, noting that the *‘ability for student to stay for whole day; [being] able to attend meetings and visits which are not part of usual teaching visits was very positive’*. Others note that the sense that ACE activities are not tied to specific learning objectives (unlike lectures for example) makes the experience more flexible and creates less pressure for encounters with patients/clients to follow a prescriptive agenda ‘*It was so nice to offer community experience without having to tick boxes and agendas for students, just to show them the interesting people they will meet as future doctors.’*

Feedback from students reflected some similar issues. Some pointed to the flexible and novel approach to both organization and content of learning creating space for knowledge acquisition. For example, one noted, ‘*A great opportunity to learn a lot of new things that I may never have even thought about.’. *Similarly, the sense of freedom from the normal curriculum was noted here: *‘A great insight into the real world of medicine, outside the closed-off work-orientated world of medical school – this week provided me with interesting medicine and stimulating work.’ *And again by this student,* ‘The week provided valuable insight into areas of medicine that we do not normally get the opportunity to explore due to the nature of our busy timetable.’ *Finally, some students used the time to contextualize learning from elsewhere in the curriculum. For example, ‘*I enjoyed the consolidation that the week brought to learning.’*

Based on the most popular specialties and community service placements, hospitals and organizations were approached to request additional places to be made available. Many students also requested individual placements or specialties via year 1 and year 2 ACE week student representatives, who forwarded these responses onto the ACE week working committee.

## ACE week: community context and partnership

Many of the opportunities made available to students through ACE week were a result of the relationships created with the community placement providers – organizations which also offer students extended placements during Phase 1. The events run by these organizations help to increase students’ awareness of the informal healthcare sector and understanding of the important function that they serve within the local community. The off-campus activities allowed students to learn about the roles of various organizations and this foundation of knowledge has allowed them to integrate their understanding with everyday clinical practice.

Following their ACE week experiences, students have taken the initiative to organize more extracurricular talks and community-led services have offered more student placements for the community placement programme. These extended links have provided some students with additional time in hospital operating theatres and time with clinicians in clinics, as well as learning from patients and practitioners. Many community-led workshops demonstrated how different professionals with global views can collaborate on their ideas and present these to students for future practice [8].

## A personal reflection

Some students have developed their learning further, practising some of the skills that they have acquired and sharing their experiences with others. For example, the lead author of this paper took part as a student in two off-campus visits (clinical placements) and on-campus activities including talks and clinical workshops during ACE week 2012. In ACE week 2013, he built on this and organized a student-led session in which those who had won prizes for project-related assignments reflected on their experiences and discussed these with their peers. Later the same year, he went on to lead a workshop about ACE week at a regional University ‘Learning and Teaching’ conference and also to present the ACE Model in London at the Centre For Advancement of Interprofessional Education (CAIPE), highlighting how students learnt from, with and about other professionals. He has gone on to undertake further research into learning methods used by medical students and presented these at an international conference.

## A platform for progression

The increased clinical exposure during Phase 1 has helped students prepare for clinical years and specific patient-based workshops provide a platform for learning styles in years 3 and 5. Workshops like these allowed students to practise their communication and examination skills with patients, rather than on each other or in simulated clinical teaching sessions.

## Theory in practice

Working with staff with extended roles and being involved in practice enabled students to witness the importance and effectiveness of inter-professional education [2]. The programme reflected aspects of the Facilitated Collaborative Interprofessional Learning model, which combines three different pedagogies: guided discovery learning, collaborative learning and interprofessional learning [9]. ACE week has been able to provide these powerful opportunities and therefore now forms an integral part of the Phase 1 curriculum.

Importantly, this model of integrated teaching, with increased clinical exposure, can be used more widely in healthcare programmes. Once strong relationships have formed between the university and external organizations, it appears that they are willing to get involved and share valuable learning experiences with students. This effective mutualistic relationship provides students with opportunities to learn and understand the important role that organizations have within the community.

## Conclusion

This integral learning intervention demonstrates how students can gain valuable experience and skills by working with external organizations. This allows students to understand and develop interprofessional relationships and become better prepared with additional experiences that can be utilized as they progress through the clinical years. Having established its acceptability and feasibility, we are excited by the prospect of developing evaluation which captures more detail about the impact of this student experience on their performance in areas such as communication and clinical skills, and understanding of the social basis and context of medicine. The ACE week is a simple demonstration of how curriculums can enjoy success within their communities, which has potential to be replicated across healthcare education programmes.
